# Diagnostic value of plasma heparin-binding protein and the heparin-binding protein-to-albumin ratio in patients with community-acquired Pneumonia: a retrospective study

**DOI:** 10.1186/s12879-023-08762-3

**Published:** 2023-11-09

**Authors:** Xueqin Xiao, Yiyu Hong, Shuo Wang, Mingliu Ma, Zhaozhong Xu

**Affiliations:** 1 Department of Emergency, Zhujiang Hospital, Southern Medical University, Guangzhou, China; 2 Department of General Practice, Sun Yat-sen Memorial Hospital, Sun Yat-sen University, Guangzhou, China

**Keywords:** Community acquired Pneumonia, Heparin-binding protein

## Abstract

**Background:**

Patients presenting to the emergency department with community-acquired pneumonia (CAP) are characterized by advanced age, comorbidities, critical illness and less-than-typical symptoms, posing a diagnostic challenge. Plasma heparin-binding protein (HBP) and the heparin-binding protein-to-albumin ratio (HBP/Alb) have not been adequately studied in the early diagnosis of CAP. This study assessed the diagnostic value of plasma HBP, HBP/Alb, and conventional inflammatory markers in emergency department patients with CAP.

**Methods:**

We enrolled 103 patients with CAP, retrospectively analyzed the patients’ clinical data, and divided the CAP patients into antibiotic (n = 79) and non-antibiotic (n = 24) groups based on whether antibiotics were administered prior to blood sampling and laboratory tests. The control group was comprised of 52 non-infected patients admitted during the same period. Within 24 h of admission, plasma HBP, serum procalcitonin (PCT), white blood cell count (WBC), neutrophil-to-lymphocyte ratio (NLR) and HBP/Alb levels were collected separately and compared. The receiver operating characteristic (ROC) curve was plotted to assess the diagnostic value of each indicator for CAP patients. Utilizing the Kappa test, the consistency of each indicator used to evaluate CAP and clinical diagnosis was analyzed. Spearman correlation was used to analyze the correlation between plasma HBP and clinical indicators of CAP patients.

**Results:**

Plasma HBP, serum PCT, WBC, NLR and HBP/Alb were all elevated in the CAP group in comparison to the control group (*P* < 0.001). Plasma HBP, serum PCT, WBC, NLR and HBP/Alb levels did not differ statistically between antibiotic and non-antibiotic groups (*P* > 0.05). Plasma HBP and HBP/Alb had the highest diagnostic accuracy for CAP, the area under the ROC curve (AUC) were 0.931 and 0.938 (*P* < 0.0001), and the best cut-off values were 35.40 ng/mL and 0.87, respectively. In evaluating the consistency between CAP and clinical diagnosis, the Kappa values for HBP, PCT, WBC, NLR and HBP/Alb were 0.749, 0.465, 0.439, 0.566 and 0.773, respectively. Spearman correlation analysis showed that plasma HBP was positively correlated with serum PCT, WBC, NLR and HBP/Alb in CAP patients (*P* < 0.001).

**Conclusions:**

Plasma HBP and HBP/Alb have a high clinical diagnostic value for CAP and can be used as good and reliable novel inflammatory markers in the emergency department for the early diagnosis of CAP patients.

## Background

Community-acquired pneumonia (CAP) is the third leading cause of death globally and one of the most prevalent infectious diseases in emergency departments [1, 2]. The Global Burden of Disease Study reported that lower respiratory tract infection is the fourth leading cause of death and years of life lost in 2019 [3]. CAP is defined as extrahospital infectious parenchymal inflammation of the lung [4]. Currently, the clinical diagnosis of CAP is mainly based on symptoms, signs, and chest imaging findings, and there is no method for rapidly obtaining pathogenic findings.

Emergency department patients with CAP are characterized by their advanced age, multiple comorbidities, critical condition and unusual symptoms [5, 6]. Approximately 10% of inpatients require intensive care support, particularly among the elderly and pre-morbid CAP patients [1]. Early diagnosis and accurate treatment of CAP are essential for clinicians to improve patient prognosis, with early diagnosis being the key to reducing hospitalization and mortality rates. Consequently, the search for ideal biomarkers for the early diagnosis of CAP is crucial for guiding the clinical management of CAP.

Heparin-binding protein (HBP) is a multifunctional protein that is pre-prepared and stored primarily in the azurophilic granules and secretory vesicles of neutrophils [7]. HBP functions include antibacterial effects, chemotaxis of monocytes and lymphocytes, and the induction of vascular leakage [8]. When there is an infection or inflammation, neutrophils are activated and rapidly release HBP [9]. As a novel inflammatory marker, HBP was widely applied in clinical practice for sepsis, interstitial lung disease, urinary tract infections, and meningitis or ventriculitis [10–13]. However, the role of plasma HBP in the early diagnosis of CAP has not been thoroughly investigated.

Russell et al. proposed and applied the heparin-binding protein-to-albumin ratio (HBP/Alb) to predict the risk of acute kidney injury in septic shock for the first time in 2018 [14]. HBP/Alb is a novel inflammatory marker derived from a simple ratio of plasma HBP to serum albumin. This index has not been reported in CAP, and its potential clinical application requires additional exploration.

In the present study, we quantified plasma HBP, serum procalcitonin (PCT), white blood cell count (WBC), neutrophil-to-lymphocyte ratio (NLR) and HBP/Alb levels in patients with CAP. The early diagnostic value of plasma HBP, HBP/Alb, and conventional inflammatory markers in patients with CAP in the emergency department was explored.

## Methods

### Study population

The study population was comprised of 103 CAP patients admitted to the emergency department of Zhujiang Hospital, Southern Medical University, from January 2021 to August 2022. Patients with CAP were divided into antibiotic group (n = 79) and non-antibiotic group (n = 24) based on whether or not they used antibiotics prior to hospital admission for blood sampling and laboratory tests. Inclusion criteria: (1) community-onset, age ≥ 18 years old; (2) lung imaging indicative of pulmonary infection symptoms; (3) fulfillment of any of the following clinical signs associated with pneumonia: recently developed respiratory symptoms, including cough, sputum production, or worsening of symptoms associated with pre-existing respiratory disease, with or without purulent sputum, chest pain, dyspnea, and hemoptysis; fever; indications of solid lung lesions and/or wet rales; WBC > 10×${10}^{9}$/L or < 4×${10}^{9}$/L [15]. Exclusion criteria: combination of other infectious diseases; history of major surgery and trauma in the last 2 weeks; severe liver and kidney insufficiency; long-term use of antibiotics and immunosuppressive drugs; combination of cardiovascular diseases such as acute myocardial infarction, myocardial ischemia and myocarditis; combination of hematological diseases such as granulocyte deficiency, aplastic anemia and acute leukemia; combination of pulmonary lesions caused by autoimmune diseases such as systemic lupus erythematosus and dry syndrome; presence of life-threatening primary diseases, such as acute phase of cerebral hemorrhage and acquired immunodeficiency syndrome.

The control group consisted of 52 non-infected patients admitted to the emergency department during the same period. During their hospitalisation, patients in the control group did not develop infections or acute coronary syndrome.

The present study was performed in accordance with the principles of the Declaration of Helsinki, and approved by the Ethics Committee of Zhujiang Hospital, Southern Medical University (2022-KY-258-02).

### Data collection

The following clinical data were collected from the medical records of enrolled patients within 24 h of admission: age, gender, systolic pressure, diastolic pressure; plasma HBP, serum PCT, WBC, neutrophil count and percentage, lymphocyte count and percentage, albumin, alanine aminotransferase (ALT), aspartate aminotransferase (AST), blood urea nitrogen (BUN), creatinine (Cr), D-dimer (DDI), and calculation of NLR, HBP/Alb for each patient. Additionally, pertinent patient history, hospital stays and clinical comorbidities were collected.

### Blood tests

HBP concentration was determined using the immunofluorescence dry quantitative method with the aid of a Jet-iStar 1000 dry fluorescence immunoassay analyzer manufactured by Zhonghan Shengtai Biotechnology Co. Ltd. and the corresponding reagent kits. PCT concentration was determined using fluorescence immunoluminescence technology with a VIDAS PC full-automatic fluorescence immunoassay analyzer manufactured by Mérieux Company and the aforementioned reagent kits. The blood routine series utilized the WBC, neutrophil and lymphocyte as indicators. A hematology analyzer was employed for detection, with the Myriad BC6000 plus automatic hematology analyzer and the appropriate supporting reagents serving as the measurement device. A BS2000M biochemistry instrument and reagent packages manufactured by Shenzhen Myriad Bio-medical Electronics Co. Ltd. were utilized to determine the albumin concentration via immunoturbidimetric means. The laboratory of the hospital performed routine analyses for ALT, AST, BUN, Cr and DDI concentrations.

### Statistical analysis

Normally distributed data were shown as mean ± standard deviation and t-test was used for comparison between groups. Non-normally distributed data were expressed as median and interquartile range (IQR), using the Mann-Whitney U test for comparison between groups. The enumeration data were shown as counts (percentages) and Fisher’s exact probability test was used. The receiver operating characteristic (ROC) curve was used to assess the diagnostic performance of each index for CAP, and the area under the ROC curve (AUC) was calculated to determine the best cut-off value. Using the Kappa test, the consistency of each indicator used to evaluate CAP and clinical diagnosis was analyzed. Spearman correlation analysis was used to examine the relationship between HBP and various clinical indicators in CAP patients. The results were deemed significant if the p-values were ≤ 0.05. All statistical analyses were conducted using IBM SPSS 25, MedCalc 20, and GraphPad Prism 8 software.

## Results

### Demographics and clinical characteristics

A total of 103 CAP patients and 52 controls were included in this study. The specific clinical information is presented in Table 1. There were no statistically significant differences between the two groups in terms of gender, age, systolic and diastolic pressure, liver or renal function. Comorbidities were common among the enrolled patients, and there were no significant differences in comorbidities between the two groups. The median length of hospital stays for CAP patients was 6 days, which was longer than for the control group (*P* = 0.006).

Compared to the control group, the levels of HBP, PCT, WBC, neutrophil count and percentage, NLR, HBP/Alb and DDI were statistically significantly higher in the CAP group, while the levels of lymphocyte count and percentage as well as albumin were statistically significantly lower (*P* < 0.001). The detailed results are presented in Table 1.

### Levels of inflammatory markers between antibiotic and non-antibiotic groups

**Fig. 1:**
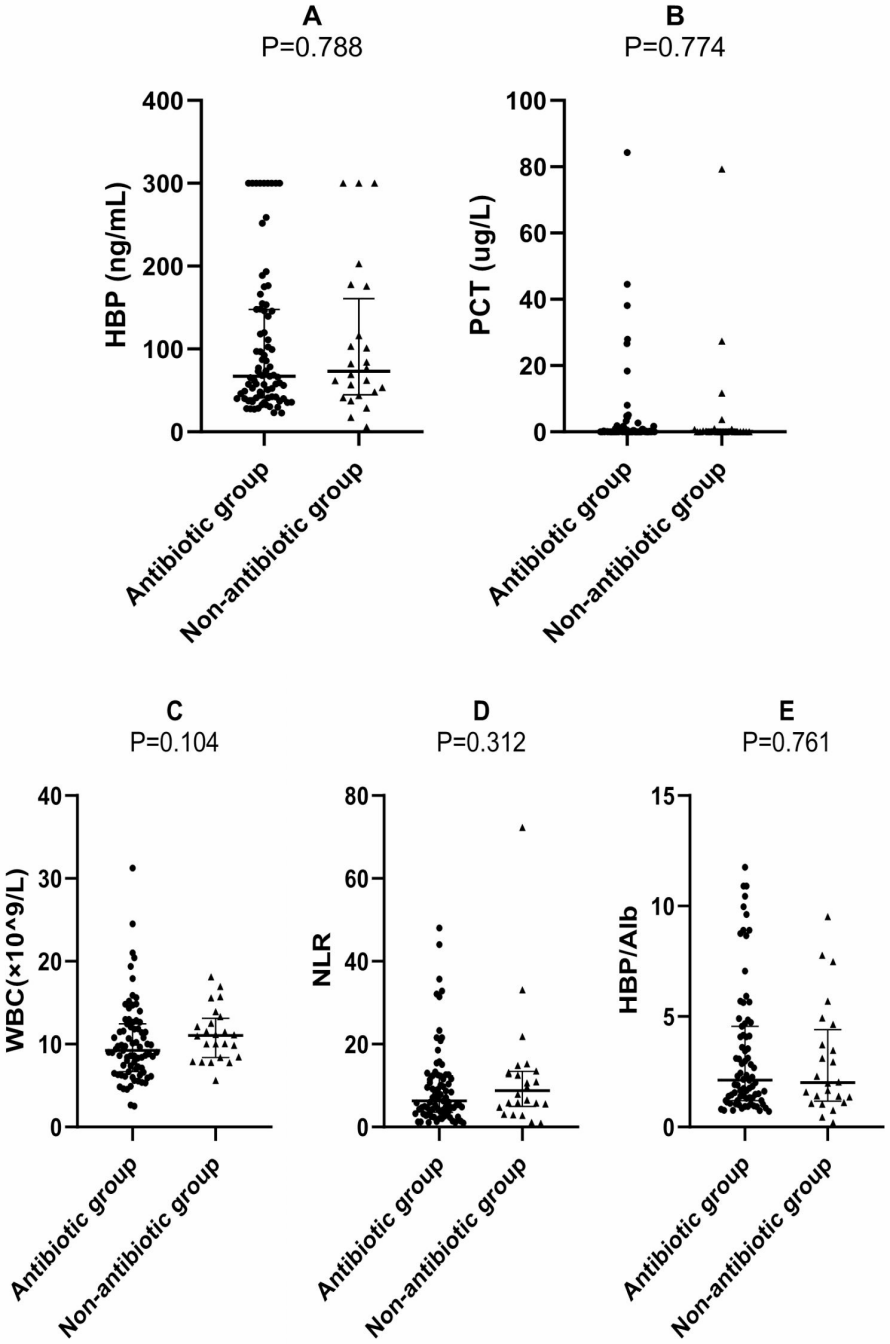
Levels of each inflammatory marker between the antibiotic and non-antibiotic groups

Patients in the antibiotic group had lower levels of HBP, PCT, WBC and NLR than those in the non-antibiotic group, while HBP/Alb levels were elevated. However, the differences in the levels of all inflammatory markers were not statistically significant between the two groups (*P* > 0.05) (Table 2; Fig. 1).

**Table 1:** Demographics and clinical characteristics between the CAP and control groups

Variable	CAP (n = 103)	Control(n = 52)	P-values
Gender, n (%) male	60(58.3)	23(44.2)	0.125
Age(years)	73.0(64.0,83.5)	68.0(59.0,77.5)	0.096
Systolic pressure(mmHg)	131.4 ± 20.28	132.9 ± 18.97	0.658
Diastolic pressure(mmHg)	76.0(67.5,86.0)	78.0(71.5,87.5)	0.266
Comorbidities, n (%)			
Hypertension	56(54.4)	34(65.4)	0.229
Diabetes mellitus	29(28.2)	18(34.6)	0.461
Malignancy	7(6.8)	2(3.8)	0.719
Kidney disease	11(10.7)	4(7.7)	0.775
ALT(IU/L)	15.0(9.0,23.0)	19.5(11.5,27.0)	0.125
AST(IU/L)	20.0(15.0,27.0)	18.0(15.0,26.0)	0.425
BUN (mmol/L)	5.27(3.80,7.55)	4.91(4.02,6.30)	0.612
Cr(umol/L)	76.0(64.5,93.5)	73.0(62.5,89.0)	0.535
Hospital stays(days)	6(5,7)	5(4,6)	0.006
HBP (ng/mL)	68.28(41.62,146.92)	16.18(11.46,27.81)	< 0.001
PCT (ug/L)	0.08(0.05,0.63)	0.05(0.05,0.05)	< 0.001
WBC(×${10}^{9}$/L)	9.85(7.60,12.49)	6.64(5.51,8.00)	< 0.001
Neutrophil(×${10}^{9}$/L)	7.01(5.25,10.50)	3.70(3.00,5.56)	< 0.001
Lymphocyte(×${10}^{9}$/L)	1.06(0.74,1.56)	1.80(1.29,2.34)	< 0.001
NLR	6.97(3.72,12.58)	2.35(1.43,3.52)	< 0.001
Neutrophil (%)	81.1(68.3,87.5)	62.3(51.6,69.0)	< 0.001
Lymphocyte (%)	12.1(6.9,18.9)	26.7(19.5,35.9)	< 0.001
Albumin (g/L)	33.9 ± 5.57	37.6 ± 3.96	< 0.001
HBP/Alb	2.03(1.24,4.36)	0.43(0.29,0.77)	< 0.001
DDI (mg/L)	1.00(0.54,2.32)	0.58(0.31,1.07)	< 0.001

**Table 2:** Basic characteristics and levels of inflammatory markers between the antibiotic and non-antibiotic groups

Variable	Antibiotic group (n = 79)	Non-antibiotic group (n = 24)	P-values
Gender,n (%) male	48(60.8)	12(50.0)	0.357
Age(years)	72.0(60.0,83.0)	77.5(68.0,84.5)	0.062
Hospital stays(days)	6(5,7)	6(5,8)	0.207
HBP (ng/mL)	67.02(41.13,146.92)	73.01(46.00,145.98)	0.788
PCT (ug/L)	0.08(0.05,0.55)	0.10(0.05,0.75)	0.774
WBC(×${10}^{9}$/L)	9.24(6.85,12.27)	11.05(8.41,12.92)	0.104
NLR	6.30(3.61,12.34)	8.76(5.16,13.35)	0.312
HBP/Alb	2.12(1.24,4.36)	2.01(1.23,4.18)	0.761

### ROC curve analysis of inflammatory markers for distinguishing CAP from control group

**Fig. 2:**
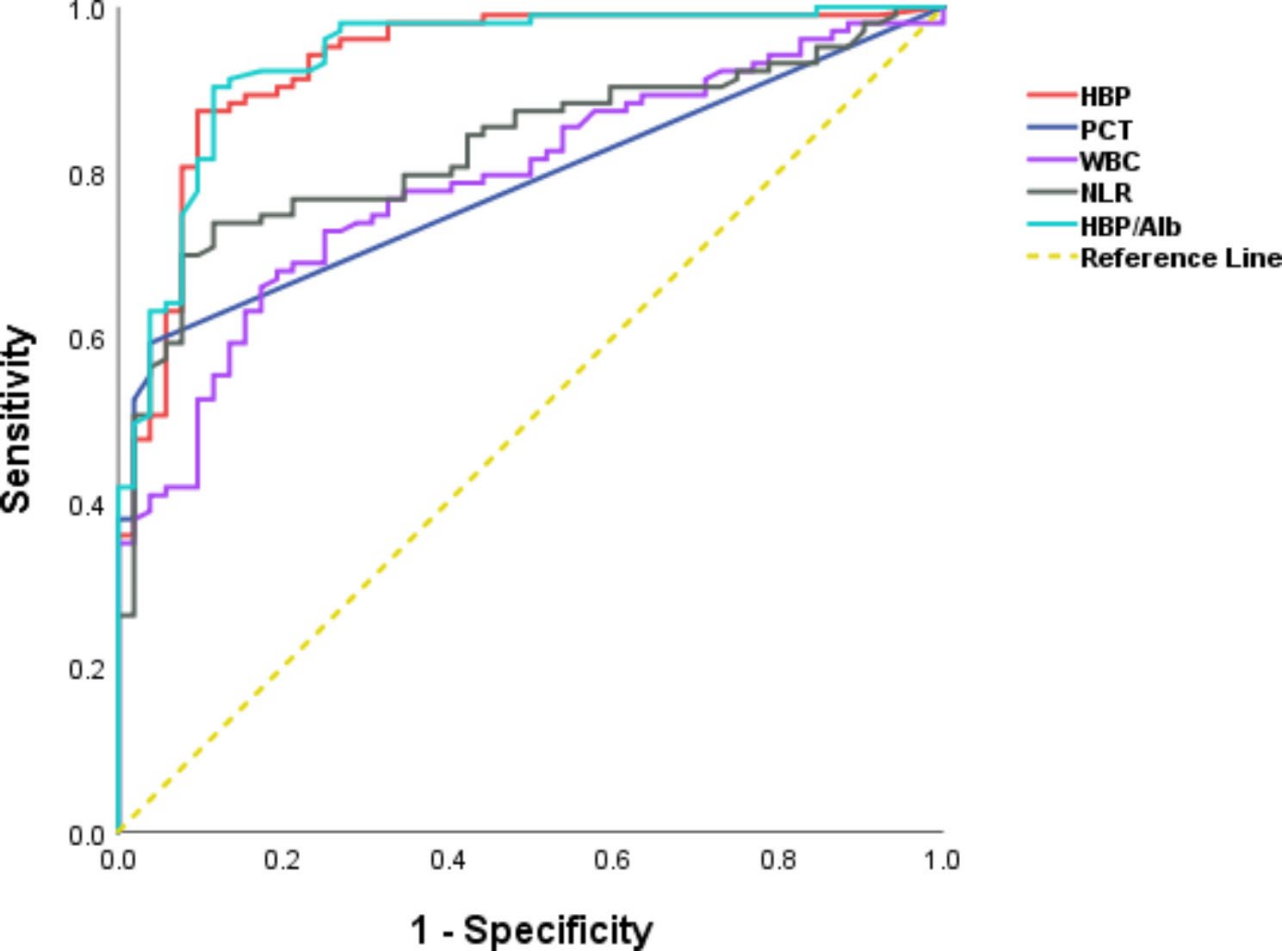
ROC curve analysis of inflammatory markers

Figure 2 displays the ROC curves of the diagnostic efficacy of each inflammatory marker for CAP. The results of the study showed that the AUCs of HBP, PCT, WBC, NLR and HBP/Alb in identifying patients in the CAP group and non-infected controls were 0.931, 0.783, 0.787, 0.829 and 0.938, respectively (Table 3). HBP and HBP/Alb had greater diagnostic value than traditional inflammatory markers such as PCT, WBC and NLR, and the best cut-off values for HBP and HBP/Alb were 35.40 ng/mL and 0.87, respectively (Table 3).

**Table 3:** Comparison of ROC curve parameters of various indicators for CAP

Indicators	AUC (95%CI)	Best cut-off value	P-values	Sensitivity (%)	Specificity (%)
HBP	0.931(0.887–0.975)	35.40ng/mL	< 0.0001	87.38	90.38
PCT	0.783(0.713–0.853)	0.05ug/L	< 0.0001	59.22	96.15
WBC	0.787(0.716–0.858)	8.36×${10}^{9}$/L	< 0.0001	67.96	80.77
NLR	0.829(0.765–0.893)	4.15	< 0.0001	73.79	88.46
HBP/Alb	0.938(0.897–0.978)	0.87	< 0.0001	90.29	88.46

CI: confidence interval

### Consistency of inflammatory markers in evaluating CAP and clinical diagnosis

Using the best cut-off values derived above, HBP > 35.40ng/mL, PCT > 0.05ug/L, WBC > 8.36×${10}^{9}$/L, NLR > 4.15, and HBP/Alb > 0.87 were considered positive for early diagnosis of CAP, as expressed in Table 4. Generally speaking, when Kappa ≥ 0.7, it indicated a strong match; when Kappa was between 0.4 and 0.7, it indicated an average match; when Kappa < 0.4, it indicated a weak match. The Kappa test values for early assessment of CAP and clinical diagnostic concordance for HBP, PCT, WBC, NLR and HBP/Alb were 0.749, 0.465, 0.439, 0.566 and 0.773, respectively (*P* < 0.001). HBP and HBP/Alb had better concordance with clinical diagnosis. The sensitivity of HBP and HBP/Alb for early diagnosis of CAP was 87.38% and 90.29%, the specificity was 90.38% and 88.46%, the positive predictive value (PPV) was 94.74% and 93.94%, and the negative predictive value (NPV) was 78.33% and 82.14%, respectively.

**Table 4:** Kappa values for HBP, PCT, WBC, NLR and HBP/Alb

Diagnostic biomarker		Clinical diagnosis		Total	Kappa
		Positive	Negative		
HBP	Positive	90	5	95	0.749
	Negative	13	47	60	
PCT	Positive	61	2	63	0.465
	Negative	42	50	92	
WBC	Positive	70	10	80	0.439
	Negative	33	42	75	
NLR	Positive	76	6	82	0.566
	Negative	27	46	73	
HBP/Alb	Positive	93	6	99	0.773
	Negative	10	46	56	
Total		103	52	155	

### Correlations of plasma HBP with clinical indicators in CAP patients

Spearman correlation analysis showed that plasma HBP was positively correlated with serum PCT (r = 0.375, *P* < 0.001), WBC (r = 0.428, *P* < 0.001), neutrophil (r = 0.447, *P* < 0.001), NLR (r = 0.341, *P* < 0.001) and HBP/Alb (r = 0.974, *P* < 0.001) in CAP patients. There was no correlation between plasma HBP and lymphocyte, albumin, DDI, ALT, AST, BUN, Cr, patient age, or length of hospital stays in CAP patients (*P* > 0.05).

## Discussion

In many countries, CAP has become one of the leading causes of death among emergency department patients [2]. Based on the fact that CAP patients seen in the emergency department also have an older age, more underlying diseases, critical conditions and atypical symptoms, this poses a challenge for the early diagnosis of CAP. Therefore, there is an urgent need to discover new biomarkers with the aim of achieving early diagnosis of CAP and reducing hospitalization and mortality rates. The present study has certain advantages. We studied the concentrations of various inflammatory markers in the blood of patients with CAP versus non-infected patients in the emergency department. This is the first clinical study to apply the HBP/Alb ratio as a diagnostic indicator for CAP. In addition, this is the first study to investigate in depth the effect of prephlebotomy antibiotics on various blood inflammation markers in emergency department patients with CAP.

The results of the study showed that the blood levels of all inflammatory markers were significantly higher in the CAP group than in the non-infected group. HBP and HBP/Alb had the best diagnostic efficacy and were more congruent with clinical diagnosis in the early diagnosis of CAP. Most previous studies failed to exclude the effect of prephlebotomy antibiotics on diverse inflammatory markers [16, 17]. Before their initial consultation, the majority of CAP patients in the emergency department may have self-administered or been given antimicrobials elsewhere. Therefore, it is essential to clarify the effect of antimicrobial drugs on various inflammatory blood indicators and the reliability of these inflammatory blood indicators as CAP diagnostic markers. We divided the CAP patients into antibiotic and non-antibiotic groups by retrospectively reviewing the patients’ medical orders and records before the blood tests were drawn. The use of antibiotics prior to blood sampling after hospital admission could reduce the levels of infection indicators such as HBP, PCT, WBC and NLR in the blood of CAP patients to some extent, but the differences between antibiotic and non-antibiotic groups were not statistically significant. The results indicated that a brief period and a single dosage of antibiotics had little influence on any infection indicators in CAP patients, further validating that HBP and HBP/Alb were reliable biomarkers for the early diagnosis of CAP patients in the emergency department.

A previous study demonstrated that HBP was one of the most effective diagnostic indicators for distinguishing lung infection from rejection in lung transplant recipients [18]. Our data showed that plasma HBP was significantly elevated in CAP patients, with an optimal cut-off value of 35.40 ng/mL, which was higher than the previous study’s result of 15 ng/mL [19]. However, unlike previous studies [19, 20], the present study population differed in a number of ways. The control group was comprised of non-infected patients admitted to the emergency department during the same period. The median plasma HBP level in these control cases was 16.18 ng/mL, presumably due to the older age, complexity and multiple comorbidities of the patients who presented to the emergency department. The expression of HBP is higher among patients with cardiovascular diseases, such as atherosclerosis, myocarditis, myocardial infarction and myocardial ischemia than in normal hearts [21]. Nevertheless, we found no significant difference in the proportion of comorbidities between the groups, and patients combined with cardiovascular diseases including acute myocardial infarction, myocardial ischemia and myocarditis had been excluded from the nadir criteria. Hence, we consider this HBP cut-off value to be more representative in the clinical management of CAP in the emergency department.

In response to an infection, neutrophils in the circulating blood become activated and release HBP, resulting in an increase in the permeability of vascular endothelial cells [22]. Albumin has been reported to prevent the HBP-induced increase in endothelial cell permeability by binding to the endothelium via the glycocalyx [14, 23, 24]. HBP/Alb was first utilized in the clinical evaluation of CAP as a novel diagnostic marker. This study showed a strong correlation between HBP/Alb and plasma HBP, as well as a higher diagnostic efficacy for the early diagnosis of CAP. This result was consistent with the conclusion of a previous study that plasma HBP levels and the HBP/Alb ratio were significant biomarkers of inflammatory response and organ dysfunction in septic shock patients [14].

In the present study, we discovered that an elevated HBP level at admission was not significantly correlated with the length of hospital stays in CAP patients and could not be used to predict patient prognosis. Conversely, other investigations demonstrated that high HBP was strongly related with a bad outcome in CAP patients [25, 26]. Different definitions of prognosis in different studies may account for this divergence, whereas in this analysis prognosis was defined as length of hospital stays.

There were several limitations to our study. Firstly, we were a single-center retrospective study. Secondly, the number of CAP cases in the non-antibiotic group was small. Thirdly, only a single point was analyzed for each blood inflammatory marker. Future large prospective studies may require dynamic monitoring of plasma HBP, serum PCT, WBC, NLR and HBP/Alb to assess the predictive value of these indicators for the risk of disease progression and death in CAP patients.

## Conclusions

This study provides evidence that plasma HBP and HBP/Alb are viable inflammatory markers for the early diagnosis of CAP patients in the emergency department. Furthermore, their diagnostic efficacy surpasses that of the conventional markers PCT, WBC and NLR. Besides, it should be noted that the utilization of antibiotics prior to sampling blood does not appear to have an impact on the dependability of inflammatory markers including HBP, PCT, WBC, NLR and HBP/Alb for the evaluation of CAP.

## Data Availability

The datasets generated and/or analyzed for the present study are not publicly available due to limitations on the ethics approval, which involves patient data and anonymity. However, these may be made available from the corresponding author on reasonable request.

## Abbreviations

CAP: Community acquired pneumonia
HBP: Heparin-binding protein
HBP/Alb: Heparin-binding protein-to-albumin ratio
PCT: Procalcitonin
WBC: White blood cell count
NLR: Neutrophil-to-lymphocyte ratio
ROC: Receiver operating characteristic curve
AUC: Area under the curve
ALT: Alanine aminotransferase
AST: Aspartate aminotransferase
BUN: Blood urea nitrogen
Cr: Creatinine
DDI: D-dimer
CI: Confidence interval
